# Individualised aspiration dynamics: Calculation by proofs

**DOI:** 10.1371/journal.pcbi.1006035

**Published:** 2018-09-25

**Authors:** Bin Wu, Lei Zhou

**Affiliations:** 1 School of Sciences, Beijing University of Posts and Telecommunications, Beijing, China; 2 Center for Systems and Control, College of Engineering, Peking University, Beijing, China; 3 Department of Ecology and Evolutionary Biology, Princeton University, Princeton, New Jersey, United States of America; Instituto Superior Técnico, Univesidade de Lisboa, PORTUGAL

## Abstract

Cooperation is key for the evolution of biological systems ranging from bacteria communities to human societies. Evolutionary processes can dramatically alter the cooperation level. Evolutionary processes are typically of two classes: comparison based and self-evaluation based. The fate of cooperation is extremely sensitive to the details of comparison based processes. For self-evaluation processes, however, it is still unclear whether the sensitivity remains. We concentrate on a class of self-evaluation processes based on aspiration, where all the individuals adjust behaviors based on their own aspirations. We prove that the evolutionary outcome with heterogeneous aspirations is the same as that of the homogeneous one for regular networks under weak selection limit. Simulation results further suggest that it is also valid for general networks across various distributions of personalised aspirations. Our result clearly indicates that self-evaluation processes are robust in contrast with comparison based rules. In addition, our result greatly simplifies the calculation of the aspiration dynamics, which is computationally expensive.

## Author summary

Cooperation is the cornerstone to understand how biological systems evolve. Previous studies have shown that cooperation is sensitive to the details of evolutionary processes, even if all the individuals update strategies in the same way. Here we propose a class of updating rules driven by self-evaluation, where each individual has its personal aspiration. The evolutionary outcome is the same as if all the individuals adopt the same aspiration for regular networks, provided the selection intensity is weak enough. In addition, we provide a simple numerical method to identify the favored strategy. Our result shows a very robust class of strategy updating rules. And it implies that complexity in updating rules does not necessarily lead to the sensitivity of evolutionary outcomes.

## Introduction

Cooperation is forgoing the focal individual’s own interest to benefit others. It is ubiquitous across every level of biological systems. Yet cooperation seemingly contradicts the evolution theory, since free riders, who contribute nothing, are better off than cooperators in fitness. The gap has attracted considerable attention to tackle how and why cooperation evolves [1].

A pairwise game, in which there are only two players, is widely employed to model the conflicts between cooperation and defection. The well-known Prisoners’ Dilemma is a pairwise game [2]. While the dyadic games capture the conflicts between cooperators and defectors, they neglect that the interactions in the real world usually involve more than two individuals. For example, all the nations are involved to reduce the emission of greenhouse gases, leading to a multiplayer collective risk dilemma [3–5]. More than two yeast cells interact to form a multicellularity when the resource is rare, resulting in a multiplayer game [6]. Thus, it is natural to adopt multiplayer games as a paradigm to study cooperation.

The last decade has seen an intensive study on the mechanisms promoting cooperation including group selection, direct reciprocity and spatial reciprocity [7–14]. In particular, spatial reciprocity attracts considerable attention with the development of network science. Spatial structure is typically described as networks, where nodes represent individuals and links social ties. Individuals on networks play games only with their neighbours. In this case, cooperators form clusters not only to invade but also to resist a population of defectors [13]. Consequently, the population structure promotes both the emergence and the stabilization of cooperation [15]. Yet the evolutionary outcome can be dramatically altered on networks, even if some detail of the evolutionary process changes: in degree-homogeneous networks, Death-birth process can promote cooperation for the Prisoners’ Dilemma whereas Birth-death process cannot [13]; In degree-heterogeneous networks, the fate of cooperation relies on the rate at which interactions occur and how interactions transform into the fitness of strategies [16]. In fact, the evolutionary outcome is not robust for different evolutionary processes even in the well-mixed population where all the individuals interact with everyone else [17, 18]. Yet, all of the above works assume that all the individuals adopt the same strategy updating rule. In reality, it is a personal trait to adjust one’s own behavior. In other words, individuals are equipped with their own updating rules, resulting in a great heterogeneity in strategy updating rule across the population. Considering that the details of evolutionary processes would greatly alter the evolutionary outcome, the fate of strategies is expected to be extremely sensitive to the heterogeneity in updating rules.

Here, we study aspiration dynamics of multiplayer games on regular networks and investigate how the heterogeneity in aspiration levels alters the evolutionary outcome. The idea of aspiration is self-evaluation: an individual is likely to keep its strategy if its aspiration is met by the payoff, and to switch otherwise. Aspiration dynamics are widely present in behavioural ecology. For example, experienced ants go back to their nest based on their own chemical trails [19] or by counting their own previous steps [20]. In our manuscript, every individual has its personal aspiration. We show that cooperation is more abundant over defection for all possible personal aspiration levels provided cooperation is advantageous for a homogeneous aspiration level. Furthermore, we analytically figure out the condition under which cooperation prevails, which is also true for all two-strategy multiplayer games. On the one hand, our result clearly shows that heterogeneity in aspiration does not alter the fate of strategies, in contrast with previous results that evolutionary outcome is sensitive to the details of the evolutionary processes [18, 21]. On the other hand, the result also provides computational savings when identifying the conditions under which one strategy is more abundant than the other.

## Models

Let us consider a two-strategy *d*-player game. An individual using strategy *A* obtains payoff *a*_*k*_ when encountering *k* coplayers using strategy *A*. And an individual using strategy *B* gets payoff *b*_*k*_ when facing *k* coplayers using strategy *A*. The number of coplayers adopting strategy *A*, i.e., *k*, ranges from 0 to *d* − 1. The game is given by the following payoff table [22]:

In a well-mixed population with finite size *N*, an individual randomly chooses *d* − 1 individuals from the entire population to play with based on Table 1. It results in a hypergeometric distribution of *d* − 1 players with respect to the number of individuals using strategy *A*. In other words, individuals interact globally in the well-mixed population. The interaction between individuals, however, may be limited by territorial constraints. Thus we concentrate on the more realistic local interaction. We assume that all the individuals are located on a regular network of size *N* with degree *d* − 1. In this case, each of the *N* individuals in the population has *d* − 1 neighbours. At each time, an individual, namely *i* ranging from 1 to *N*, is randomly selected. It plays the *d*-player game with its *d* − 1 neighbours, and obtains payoff *π*_*i*_. The focal individual *i* is likely to switch its strategy if its current payoff *π*_*i*_ does not meet its aspiration *e*_*i*_.

**Table 1:** Payoff table.

Number of coplayers with strategy *A*	0	1	…	*k*	…	*d* − 1
*A*	*a*_0_	*a*_1_	…	*a*_*k*_	…	*a*_*d*−1_
*B*	*b*_0_	*b*_1_	…	*b*_*k*_	…	*b*_*d*−1_

We assume that every individual has its own way of switching. To this end, we allow that individual *i*, ranging from 1 to *N*, switches its strategy with a probability given by its own decision making function *g*_*i*_. Here *g*_*i*_ is a function of *β*(*e*_*i*_ − *π*_*i*_), with selection intensity *β* ≥ 0 [23]. All through our manuscript, we assume that the decision making function *g*_*i*_ is

1. differentiable on the real line with its derivative strictly positive, i.e., $g_{i}^{\prime }>0$;
2. between zero and one, i.e., 0 < *g*_*i*_ < 1.

For 1), the positive derivative of the decision making function $g_{i}^{\prime }>0$ implies that the decision making function is strictly increasing. In this case, an individual whose aspiration is not met is more likely to change its strategies. For 2), the bound between zero and one yields that *g*_*i*_ is a probability and that there is always a non-zero probability of changing strategies.

The aspiration dynamics on a network is a Markov chain. The state space of the Markov chain is *S* = {(*s*_1_, *s*_2_…, *s*_*N*_)|*s*_*i*_ ∈ {*A*, *B*}}, where *s*_*i*_ is 1 if the strategy of individual *i* is *A*, and is 0 otherwise. $\sum_{i=1}^{N}s_{i}$ is the number of individuals using strategy *A*. There is no absorbing state of the Markov chain, since the decision making functions we propose ensure that all the individuals could alter their strategies at any time. The Markov chain is ergodic, thus there is a stationary distribution *κ*_*s*_, *s* ∈ *S*. The average abundance of strategy *A* in the stationary regime is obtained by $\sum_{s\in S}\;\kappa_{s}\left(\sum_{i=1}^{N}s_{i}\right)/N$. Here *κ*_*s*_ is a function of selection intensity. We are interested in the condition under which strategy *A* is favored. In other words, we try to figure out the condition under which the average abundance of strategy *A* is more abundant than that of strategy *B*. i.e., $\sum_{s\in S}\;\kappa_{s}\left(\sum_{i=1}^{N}s_{i}\right)/N>1/2$.

(Table 2) summarizes key notations in this section. It is noteworthy that both the aspiration and the decision making function are personal traits, resulting in the heterogeneity in decision making.

**Table 2:** Notation list.

Notation	Implication
*N*	population size
*d*	group size, degree of regular network+1
*β*	selection intensity
*g*_*i*_	decision making function of individual *i*
*π*_*i*_	payoff of individual *i*
*e*_*i*_	aspiration/expectation of individual *i*
*s*_*i*_	state of individual *i*

### An equivalent condition that strategy *A* is favored

We show that the average abundance of strategy *A* is one half when the selection intensity vanishes. And this remains true for all possible individualised aspirations *e*_*i*_ and decision making functions provided *g*_*i*_(0) > 0 for all *i* = 1, 2…, *N* (see Section *The average abundance is* 1/2 *for vanishing selection intensity* in S1 File. for the proof). In particular, it is true for our decision making functions, since our decision making function *g*_*i*_ is positive for the real line. Intuitively, no individual has any bias towards any strategy for the vanishing selection intensity, although they update strategies in their own ways. Thus strategy *A* is more abundant than strategy *B* *if and only if* the abundance of strategy *A* is more than that of its neutral case, which holds for all possible individualised aspirations, aspiration-based decision making functions and selection intensities.

### The criterion is a linear inequality

In the following, we introduce two lemmas, which are crucial to the main result.

**Lemma 1** *For the decision making function g*_*i*_ *with* 0 < *g*_*i*_ < 1 *and positive derivative on the real line* $g_{i}^{\prime }>0$ (*i* = 1, 2,…, *N*), *under weak selection limit, there exist parameters α*_*k*_, *ω*_*k*_ (*k* = 0, 1, …, *d* − 1) *and ϕ*_*i*_ (*i* = 1, 2, …, *N*), *which are neither dependent on the payoff entries nor the aspiration levels, such that if*

$$\begin{array}{c} \sum_{k=0}^{d-1}\alpha_{k}a_{k}+\sum_{k=0}^{d-1}\omega_{k}b_{k}+\sum_{i=1}^{N}\phi_{i}e_{i}>0, \end{array}$$ (1)

*strategy A is more abundant than strategy B*.

We outline the proof: The differentiability of the decision making function ensures that the average abundance of strategy *A*, i.e., $\sum_{s\in S}\;\kappa_{s}\left(\sum_{i=1}^{N}s_{i}\right)/N$, is differentiable with respect to the selection intensity [24]. Thus the average abundance of strategy *A* under weak selection limit is given by

$$\begin{array}{c} \sum_{s\in S}\kappa_{s}\left(0\right)(\sum_{i=1}^{N}s_{i})/N+(\sum_{s\in S}\kappa_{s}^{\prime }\left(0\right)(\sum_{i=1}^{N}s_{i})/N)\beta +o\left(\beta \right). \end{array}$$ (2)

The first term is the average abundance of strategy *A* when the selection intensity vanishes, it is one half based on Section *The average abundance is* 1/2 *for vanishing selection intensity* in S1 File. Therefore, the average abundance of strategy *A* is more than that in the neutral case if $\sum_{s\in S}\kappa_{s}^{\prime }\left(0\right)\left(\sum_{i=1}^{N}s_{i}\right)$ is positive. Thus $\sum_{s\in S}\kappa_{s}^{\prime }\left(0\right)\left(\sum_{i=1}^{N}s_{i}\right)>0$ is the condition under which strategy *A* is more abundant than strategy *B*. We show that $\sum_{s\in S}\kappa_{s}^{\prime }\left(0\right)\left(\sum_{i=1}^{N}s_{i}\right)$ is linear in payoff and aspiration level with no constant term, i.e., a term does not contain payoff entries or aspirations (Section *The criterion is a linear inequality of payoffs and aspirations* in S1 File).

**Lemma 2** *If all the payoff entries are the same, i.e., there exists a constant h such that a*_*k*_ = *b*_*k*_ = *h* *for all k* = 0, 1…, *d* − 1, *then the average abundance of strategy A is one half for all possible individualised aspirations and all aspiration-based decision making functions fulfilling g*_*i*_(0) > 0, *i* = 1, 2, …, *N* *and all selection intensities*.

(See Section *The average abundance is* 1/2 *for neutral mutants for any selection intensity* in S1 File for the proof.)

Lemma 2 suggests that if a neutral mutant arises, the heterogeneity in the decision updating rule and aspiration level does not alter the evolutionary outcome, compared with the homogeneous case. In particular, Lemma 2 is true for any selection intensity. Intuitively, both strategies are identical in payoffs when all the payoff entries are the same. It resembles a neutral mutant in population genetics [25]. Furthermore, each individual shows no bias towards any of the two strategies, although everyone has its own way to update strategies. By symmetry, the two strategies should be equally abundant.

## Results

### The condition is independent of individualised aspirations

**Theorem 1** *Consider a regular network with degree d* − 1, *and the decision making function with* 0 < *g*_*i*_ < 1 *and positive derivative on the real line* $g_{i}^{\prime }>0$ (*i* = 1, 2, …, *N*), *in the limit of weak selection, there exist d parameters σ*_*k*_*s* (*k* = 0, 1…, *d* − 1) *which are neither dependent on payoff entries nor aspiration levels such that if*

$$\begin{array}{c} \sum_{k=0}^{d-1}\sigma_{k}\left(a_{k}-b_{d-1-k}\right)>0, \end{array}$$ (3)

*then strategy A is more abundant than strategy B*.

**Proof 1** *For the payoff table with all the entries being zero, i.e., a*_*k*_ = *b*_*k*_ = 0 *for* 0 ≤ *k* ≤ *d* − 1, *strategy A and B are equally abundant for any selection intensity by Lemma 2. Thus the two strategies are equally abundant under weak selection limit. On the one hand, strategy A is* not *more abundant than strategy B. Thus there exist N parameters ϕ*_*i*_*s* (*i* = 1, 2…, *N*) *which are neither dependent on the aspiration levels nor payoff entries such that* $\sum_{i=1}^{N}\phi_{i}e_{i}\leq 0$ *holds by Lemma 1. On the other hand, strategy B is* not *more abundant than strategy A*. *Thus* $\sum_{i=1}^{N}\phi_{i}e_{i}\geq 0$ *holds with the same argument by Lemma 1. Therefore, it yields that*

$$\begin{array}{c} \sum_{i=1}^{N}\phi_{i}e_{i}=0. \end{array}$$ (4)

*Note that the parameters ϕ*_*i*_*s*, *i* = 1, 2, …, *N*, *are independent of the aspirations levels*. *And* Eq (4) *is valid for any e*_*i*_*s* (*i* = 1, 2, …, *N*). *In particular, for any j* (1 ≤ *j* ≤ *N*), *let e*_*i*_ = *δ*_*ij*_, *where δ*_*ij*_ *is the Kronecker Delta. We have that ϕ*_*j*_ = 0 *by* Eq (4).

*Therefore, based on Lemma 1, the condition under which strategy A is more abundant than strategy B is*

$$\begin{array}{c} \sum_{k=0}^{d-1}\alpha_{k}a_{k}+\sum_{k=0}^{d-1}\omega_{k}b_{k}>0. \end{array}$$ (5)

*Switching the name of A and B, by Lemma 1, strategy B is more abundant than strategy A if*

$$\begin{array}{c} \sum_{k=0}^{d-1}\alpha_{k}b_{d-1-k}+\sum_{k=0}^{d-1}\omega_{k}a_{d-1-k}>0 \end{array}$$ (6)

*holds*.

*On the one hand, the linear combination of Inequality* (6) *is proportional to the first-order derivative of the abundance of strategy B; On the other hand, the sum of the abundance of strategy A and B is always one for any selection intensity. Thus the first-order derivative of the sum of the two abundances is zero. In other words, the first-order derivative of the abundance of strategy A is always different from that of strategy B in sign, provided neither of them is zero. Thus, strategy A is more abundant than strategy B* if and only if *Inequality* (6) *does not hold*.

*Therefore, strategy A is more abundant than strategy B if*

$$\begin{array}{c} \sum_{k=0}^{d-1}\alpha_{k}b_{d-1-k}+\sum_{k=0}^{d-1}\omega_{k}a_{d-1-k}<0 \end{array}$$ (7)

*or*

$$\begin{array}{c} \sum_{k=0}^{d-1}(-\omega_{d-1-k})a_{k}+\sum_{k=0}^{d-1}(-\alpha_{d-1-k})b_{k}>0. \end{array}$$ (8)

*Both Inequality* (5) *and* (8) *are the condition under which strategy A is more abundant than strategy B. Thus there exists a positive rescaling factor* λ > 0 *such that they are mapped to each other, i.e., α*_*k*_ = −λ*ω*_*d*−1−*k*_ *and* *ω*_*k*_ = −λ*α*_*d*−1−*k*_ *hold for* 0 ≤ *k* ≤ *d* − 1. *In particular, we have α*_0_ = −λ*ω*_*d*−1_ *and* *ω*_*d*−1_ = −λ*α*_0_. *Thus* λ^2^ = 1. *We have that* λ = 1 *since the constraint* λ > 0. *This yields that α*_*k*_ = −*ω*_*d*−1−*k*_ *for* *k* = 0, 1,…, *d* − 1. *In this way, Inequality* (5) *becomes*

$$\begin{array}{c} \sum_{k=0}^{d-1}\underset{\sigma_{k}}{\underset{︸}{\alpha_{k}}}\left(a_{k}-b_{d-1-k}\right)>0. \end{array}$$ (9)

*By Lemma 1, α*_*k*_ *is not dependent on the aspirations or the payoff entries. Therefore σ*_*k*_ *is neither dependent on the aspirations nor the payoff entries*.

With the aid of Lemma (1) and Lemma (2), we obtain the condition under which strategy *A* is more abundant than strategy *B*. Herein, Lemma 1 states that the criterion under which a strategy is more abundant than the other is determined by both payoff entries and individualised aspirations. Yet Lemma 2 implies that the criterion is not dependent on the aspiration levels at all, and it is the cornerstone of the proof.

The theorem indicates that the criterion under which strategy *A* is favored is a simple linear inequality (3). The coefficients of the inequality are neither dependent on the payoff entries nor the aspirations, but are determined by the decision making functions, the way of payoff collecting, population size and the population structure.

The coefficients of the inequality do not depend on the payoff entries, which is similar to the *σ*-rule of the mutation-selection dynamics [24, 26]. The independence ensures that the coefficient *σ*_*k*_ is obtained by adopting the payoff table with *a*_*k*_ = 1 and all the rest being zero. Thus it opens up an avenue to compute the evolutionary dynamics under weak selection limit [27].

**Fig 1:**
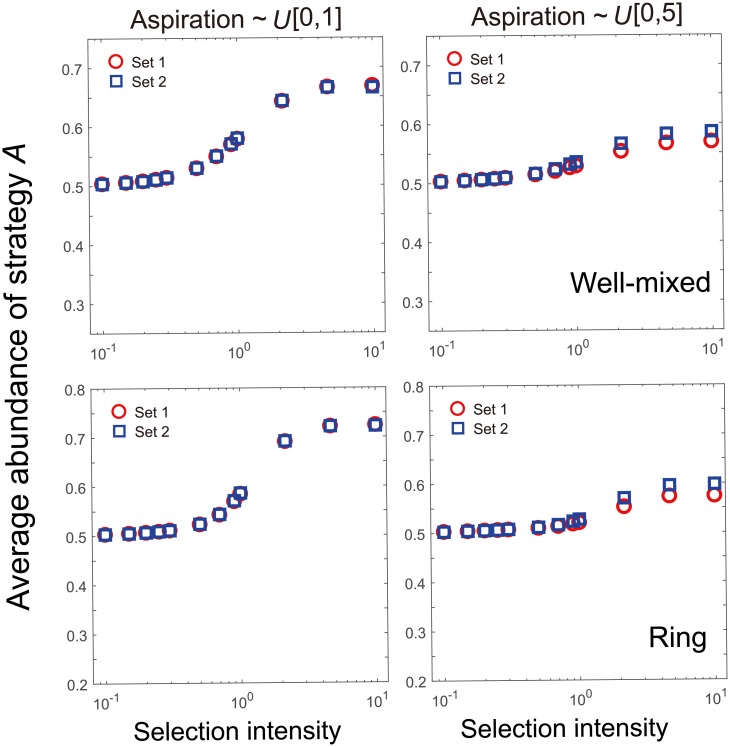
Average abundance of strategy *A* for a three-player game with individualised aspirations. Personal aspirations are randomly assigned based on a uniform distributions on the interval [0, 1] (left panels) or [0, 5] (right panels). Within each class, two sets of aspirations are assigned to represent two populations with different aspirations from the same distribution. In this way, we established two kinds of heterogeneity in aspiration: one resorts to the underlying distribution, and the other is based on the actual values sampled from the same distribution. Intuitively, both of the two heterogeneities would alter the evolutionary outcome. Simulations in line with our theorem, however, show that both kinds of the heterogeneity in aspiration lead to the identical abundance in *A* for both the well-mixed population (upper panels) and that on rings (lower panels). We illustrate other details of the simulation in the following. In well-mixed populations, the focal individual randomly chooses two individuals from the rest of the population, whereas it plays only with the nearest two neighbours on the ring. In the beginning, we randomly set 5% of the population to be of strategy *A* and the rest to be of strategy *B*. For each data point, it is the mean of 20 independent runs. In each run, we iterate the evolutionary process for 1 × 10^8^ generations. The average abundance of strategy *A* is obtained by averaging abundance of strategy *A* over the last 5 × 10^7^ generations. The population size *N* = 100. And the payoff entries are *a*_0_ = 3, *a*_1_ = 2, *a*_2_ = 1, *b*_0_ = 4, *b*_1_ = 1 and *b*_2_ = 1, respectively.

**Fig 2:**
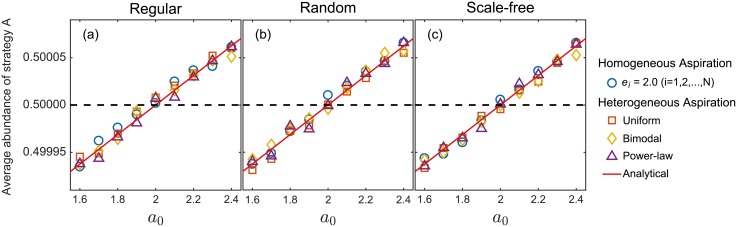
Average abundance of strategy *A* as a function of the payoff entry *a*_0_. Our simulation results clearly show that strategy *A* is more abundant than strategy *B* if *a*_0_ is approximately greater than 2.0 for regular networks (*k* = 2) (Panel (a)). This is in perfect agreement with our calculation based on the theorem, i.e., inequality *a*_0_ + 2*a*_1_ + *a*_2_ > *b*_0_ + 2*b*_1_ + *b*_2_ with *a*_1_ = 2, *a*_2_ = 1, *b*_0_ = 4, *b*_1_ = 1 and *b*_2_ = 1. Note that the criterion *a*_0_ > 2 is valid for aspiration across various distributions. It implies that the criterion to favor one strategy over the other is independent of the individualised aspiration, as stated in the theorem. Furthermore, the criterion also holds beyond regular networks (Panel (a)), namely, on random (Panel (b)) and scale-free networks (Panel (c)). It suggests that the criterion can be extrapolated to general population structures. The details of the simulations are as follows: The minimum degree of all the networks is set to be two such that all the individuals have enough neighbours to play the three-player game with. Personal aspirations are randomly assigned in a population with homogeneous aspiration *e*_*i*_ = 2 for all *i* = 1, 2…*N* (blue ◯), and a population with heterogeneous aspirations generated based on uniform distribution on the interval [0, 5] (red □), bimodal distribution with $e_{i}∼0.4N\left(2.5,0.5^{2}\right)+0.6N\left(4.5,0.5^{2}\right)$ (orange ◊), and power-law distribution with probability density function *f*(*x*) = 2*x*^−3^ (purple △). Here, $N(2.5,0.5^{2})$ stands for the normal distribution with mean 2.5 and standard deviation 0.5. In addition, the minimum value of aspiration sampled from the power-law distribution is 1.0. In the beginning, we randomly set 45% of the population to be of strategy *A* and the rest to be of strategy *B*. At each time step, the focal individual randomly chooses two individuals from its neighborhood and play a single three-player game with them to obtain the payoff. Fermi function is employed as the decision making function for all the individuals. Each data point is the mean of the average abundance of strategy *A* calculated from three independent runs (5 × 10^9^ samples in each run, 1.5 × 10^10^ samples in total). In each run, we start sampling after a relaxation time of 5 × 10^7^ time steps. The average abundance of strategy *A* is obtained by averaging the abundance of strategy *A* over 5 × 10^9^ time steps. The population size *N* = 1000. The selection intensity *β* = 0.005.

The theorem ensures that the coefficients do not depend on the aspiration levels at all. Simulations also show that the abundance of strategy *A* almost keeps invariant for a diverse class of distributions of individual aspirations (Figs (1) and (2)). It is counter-intuitive that the coefficients are independent of the aspiration levels. In fact, it is well-known for games on networks that the heterogeneity of personal traits alters the evolutionary outcome [28–32]. For example, cooperation can be greatly promoted if individuals have heterogeneous neighborhood sizes [33]. Aspirations are part of the individual’s updating rule. Thus the heterogeneity in updating rule should have changed the evolutionary outcome for some games. The intuitive understanding resorts to weak selection limit. On the one hand, the evolutionary outcome is not determined by the aspiration under weak selection limit when the aspiration is homogeneous in the population [34]. On the other hand, the driving force of the strategy updating is stochastic neutral drift under weak selection. Therefore, the effect of heterogeneity in aspiration is weakened, resulting in a similar dynamics with the case of homogeneous aspiration. In fact, individual *i* uses strategy *B* more often if the switching probability from *A* to *B* is greater than the other way around. In other words, strategy *B* is more abundant for individual *i* if *g*(*β*(*e*_*i*_ − *π*_*A*_)) > *g*(*β*(*e*_*i*_ − *π*_*B*_)), i.e., *π*_*B*_ > *π*_*A*_ under weak selection. This suggests that the criterion should be independent of the individualised aspiration, consistent with our theorem.

The coefficients are only dependent on individuals’ decision making functions, population size, the way of payoff collecting and the underlying population structure. In particular, they are determined by all the decision making functions *g*_*i*_, *i* = 1, 2⋯, *N*. Thus personalised updating rules might alter the evolutionary outcome, which is not the focus of our manuscript.

To sum up, the theorem shows that personalised updating rules might alter the evolutionary outcome, but not via heterogeneous aspiration levels.

### Computation of the criterion for one strategy to be favored over the other

So far, we have obtained the condition under which a strategy is more abundant than the other, when everyone is equipped with its own aspiration. The condition is a linear inequality with as many coefficients as the game size. Further investigations show that those coefficients are always non-negative, and at least one of them is positive (see Section *Non-negativity of the coefficients* in S1 File). It mirrors the non-negativity of the structure coefficients of the *σ*-rule for mutation-selection dynamics [35].

In the following, we estimate the *d* coefficients, i.e., *σ*_*k*_ *k* = 0, 1, 2…, *d* − 1. For simplicity, we assume that all the individuals adopt the Fermi function as the decision making function, i.e. *g*_*i*_(*x*) = [1 + exp(−*x*)]^−1^ for *i* = 1, 2⋅⋅⋅*N*.

By our theorem, the coefficients for a population with individualised aspirations are exactly the same as the coefficients for a population with the homogeneous aspiration. Therefore, the coefficients for the population with heterogeneous aspirations can be calculated via those with a homogeneous aspiration (Section *Calculation of coefficients* in S1 File). And we obtain that

$$\begin{array}{c} \sigma_{k}=\left(\begin{array}{c} d-1\\ k \end{array}\right),\;k=0,1\ldots ,d-1. \end{array}$$ (10)

Eq (10) implies that the condition under which strategy *A* is more abundant on regular networks is exactly the same as that when the population is well-mixed. Generally, Eq (10) holds, provided all the individuals have the same decision making function with non-vanishing derivative at zero. (see Section *Estimating coefficients* S1 File).

Numerically, we estimate the coefficients for a three-player game on both well-mixed populations and rings. In well-mixed populations, the focal individual gets its payoff by playing games with two coplayers randomly selected from the rest of the population while on rings, its payoff is derived from interacting with its two nearest neighbours. We set the aspirations of individuals based on a uniform distribution. In this case, the aspiration is so personal that it is of great possibility that no one shares the aspiration with anyone else. The numerical results imply that the coefficients are i) exactly the same for both well-mixed populations and rings, ii) *σ*_0_ = 1, *σ*_1_ = 2 and *σ*_2_ = 1 ((Table 3)). Therefore they are in perfect agreement with the theorem and Eq (10).

**Table 3:** Estimating the coefficients *σ*_*k*_s by simulation. We establish three sets of individualised aspirations based on the uniform distribution in [0, 1] (see S2 File for details). For three-player games with *b*_0_ = *b*_1_ = *b*_2_ = 0, the estimated coefficients *σ*_0_, *σ*_1_, *σ*_2_ are obtained by linear regression model in the form of *σ*_0_*a*_0_ + *σ*_1_*a*_1_ + *σ*_2_*a*_2_ + Intercept. For rings, the regression coefficients *σ*_0_,*σ*_1_,*σ*_2_ and Intercept are close to 1,2,1 and 0, which agrees perfectly with theoretical calculations Eq (10). In addition, it holds for all the three sets of aspirations, validating the theorem. For well-mixed populations, the *d* coefficients still hold, consistent with [34]. Thus the coefficients are robust to the heterogeneity in aspiration for both ring and well-mixed population. The confidence interval for the corresponding estimated coefficients (EC) are [EC-ME, EC+ME], where ME in the parentheses is calculated with confidence level 95%. Please refer to the Methods to see the details of the simulation. Population size *N* = 100, selection intensity *β* = 5 × 10^−2^. More details of the simulation are found in S1 File.

	Well-mixed			Ring		
	Set 1	Set 2	Set 3	Set 1	Set 2	Set 3
*σ*_0_	0.9848	0.9618	1.0027	0.9910	1.0188	1.0248
	(0.0347)	(0.0344)	(0.0338)	(0.0340)	(0.0337)	(0.0342)
*σ*_1_	1.9743	1.9453	2.0020	1.9761	2.0260	2.0526
	(0.0648)	(0.0643)	(0.0632)	(0.0634)	(0.0629)	(0.0639)
*σ*_2_	0.9941	0.9716	1.0117	0.9841	1.0140	1.0351
	(0.0347)	(0.0344)	(0.0338)	(0.0340)	(0.0337)	(0.0342)
Intercept	-0.0034	-0.0031	0.0011	0.0013	0.0019	-0.0045
	(0.0039)	(0.0039)	(0.0038)	(0.0038)	(0.0038)	(0.0039)

Simulation results, i.e., Fig (2b) and (2c) show that the criterion in the theorem to favor strategy *A* is also valid for random and scale-free networks, beyond regular networks, i.e., Fig (2a). In particular, simulations also show that the criterion is independent of individual aspirations. It is noteworthy that the number of neighbors each individual has should be no less than *d* − 1 for non-regular networks, such that everyone has enough neighbours to play the *d*-player game with.

**Fig 3:**
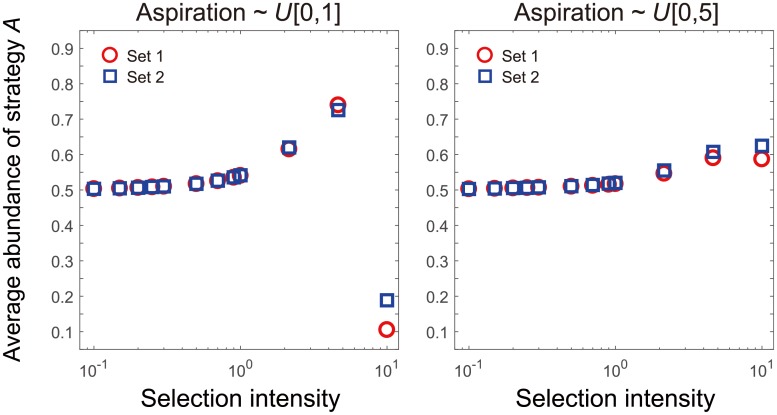
Robustness of the evolutionary outcome based on the way of payoff accumulation. Individuals are now participating in three games for a time. One of the three is organised by itself and the rest two by its two nearest neighbours. The final payoff is the average of the accumulated payoff in the three games. This payoff accumulation is different from that in the theorem, where every individual only plays a single *d*−player game. Yet we find that the heterogeneity of aspiration still does not change the abundance of strategy *A*. Thus our theorem might be generalised to other ways of payoff accumulation. Details of the simulation: The aspiration values are randomly selected from the uniform distributions on interval [0, 1] (left panel) or [0, 5] (right panel). Within each distribution, we establish two populations with different individualised aspirations. All the rest parameters are the same as those of (Fig 1).

Up till now, we assume that an individual organizes a *d*−player game with its *d* − 1 neighbours, and it gets its final payoff via this single game. We now allow this individual participates in the *d*−player games organized by thy neighbours besides the one organized by itself. In other words, the focal individual takes part in *d* such *d*−player games, one of which is organized by itself and all the rest *d* − 1 games are organized by its *d* − 1 neighbors. The final payoff of the focal individual is the average payoff over those *d* games. In this case, the focal individual’s final payoff is not only determined by its nearest neighbors, but also is up to the second nearest neighbours. We investigate whether this alternative way of payoff collection alters the criterion under which strategy *A* is more abundant. We perform simulations on rings ((Fig 3)). We find that heterogeneity in aspiration does not change the evolutionary outcome either. Thus, the simulation suggests that the way of payoff accumulation does not change our result that the evolutionary outcome is insensitive to the individualised aspirations.

Note that the theorem is obtained under the assumption of weak selection limit. It implies that the selection intensity can be sufficiently small, given the payoff entries and individual aspirations. In other words, given the payoff table and the individualised aspirations, the criterion $\sum_{k=0}^{d-1}\left(\begin{array}{c} d-1\\ k \end{array}\right)\left(a_{k}-b_{d-1-k}\right)>0$ is valid for ensuring that strategy *A* is more abundant than strategy *B*, provided the selection intensity can be tuned as small as possible. This does not mean that given a small yet fixed selection intensity and individualised aspiration, the criterion is valid. In fact, fixed selection intensity violates the assumption of the weak selection limit in our theorem. However, it brings us into a question in simulation: how weak the weak selection is to ensure that the criterion in the theorem is valid for a given individualised aspiration. The key idea is to ensure that perturbation analysis is correct. In other words, all the individuals have to be mainly driven by blind switching. And the switching probability from *A* to *B* differs little from the other way around, or the difference should be of order *β*. In other words, *g*(*β*(*e*_*i*_ − *π*_*A*_)) − *g*(*β*(*e*_*i*_ − *π*_*B*_)), i.e.,

$$\begin{array}{l} g^{\prime }\left(0\right)\left(\pi_{B}-\pi_{A}\right)\beta +\frac{1}{2}g^{\prime \prime }\left(0\right)\left(\pi_{B}-\pi_{A}\right)\left(2e_{i}-\pi_{A}-\pi_{B}\right)\beta^{2}+o\left(\beta^{2}\right)\\ =g^{\prime }\left(0\right)\left(\pi_{B}-\pi_{A}\right)\beta \left(1+\frac{g^{\prime \prime }\left(0\right)}{2g^{\prime }\left(0\right)}\left(2e_{i}-\pi_{A}-\pi_{B}\right)\beta \right)+o\left(\beta^{2}\right) \end{array}$$ (11)

should be of order *β*. It is required that $|\frac{g^{\prime \prime }\left(0\right)}{2g^{\prime }\left(0\right)}\left(2e_{i}-\pi_{A}-\pi_{B}\right)\beta |⪡1$ makes the criterion valid. In other words, if *g*′′(0) ≠ 0, a sufficient condition to ensure that the criterion is valid is that

$$\beta \ll \frac{g^{\prime }\left(0\right)}{|g^{\prime \prime }\left(0\right)|[\max\{|e_{i}\left|\right\}+\max\{|a_{k}\left|,\right|b_{k}\left|\}\right]}.$$

It suggests that the larger the maximum value of the aspiration, the weaker the selection intensity is to ensure that the criterion is valid. This is consistent with Fig 1 that aspirations generated via uniform distribution on the interval [0, 5] need weaker selection intensity to make the criterion valid than those generated via the uniform distribution on the interval [0, 1]. If *g*′′(0) = 0, then $|\frac{g^{\prime \prime }\left(0\right)}{2g^{\prime }\left(0\right)}\left(2e_{i}-\pi_{A}-\pi_{B}\right)\beta |=0⪡1$ holds. Higher order expansion of the difference has to be performed to figure out how weak the weak selection is.

## Discussion

Evolutionary processes are typically categorised into two classes, one is comparison based and the other is self-evaluation based. Comparison means that strategies are adjusted via payoff comparison. For example, imitation is comparison based. Therein, an individual compares its payoff with another one’s, and is likely to adopt the strategy of the more successful. Thus the comparison happens between a pair of individuals. Fitness-based process is also comparison based, including Moran process [36–39] (Death-birth and Birth-death) and Wright-Fisher process [40]. Therein, individuals reproduce with a probability, which is based on the payoff comparison among all the competing individuals. Individuals with higher payoffs are more likely to reproduce. In this case, the comparison occurs among all the individuals in the population. Self-evaluation indicates that an individual is likely to alter its strategy if the payoff does not meet its aspiration. It is similar to the ‘Win-stay-lose-shift’ strategy [41]. For the self-evaluation process, we do not take the cost of evaluation into account for the sake of simplicity following the convention in evolutionary game theory [42–45]. Compared with the comparison-based individuals, individuals with self-evaluation based updating rules only make use of their own information to update the strategy. In addition it introduces ‘exploration’ [46], since an entire population with all the same strategy can have a new mutant, provided an individual is not satisfied with its current payoff. It, however, is impossible for comparison-based individuals unless exploration is additionally assumed. Differences between the two classes of evolutionary processes suggest that they can lead to different evolutionary outcomes. In fact, differences arise even when the population is well-mixed and selection intensity is weak [34], where evolutionary outcomes are almost identical for all the comparison-based processes [23, 47].

Strategy updating rule, either comparison-based or self-evaluation-based, has been typically assumed to be homogeneous for all the individuals. All the individuals make decisions in the same way. Yet strategy updating rule is a personal trait and differs among individuals. We tackle how the heterogeneity in strategy updating rules alters the evolutionary outcome. Previous studies have shown that this heterogeneity could dramatically alter the evolutionary outcome, if individuals are equipped with personalised comparison-based rules [28, 29, 31]. In particular, the heterogeneity can promote cooperation in spatially structured populations. This is in line with the expectation, since the evolutionary outcome is sensitive to the details of the comparison-based processes, even if the updating rule is homogeneous [18]. However, it is still unclear what happens if the evolutionary processes are chosen in the class of self-evaluation based processes. Or how this heterogeneity affects the evolutionary outcome within the class of self-evaluation processes. Intuitively, it is natural to expect that alternative outcomes emerge if individuals are using their own self-evaluation rules. Our theorem, however, shows the opposite: the outcome based on heterogeneous aspirations is the same as that for homogeneous ones under weak selection limit. In fact, it results from the fact that the evolutionary outcome is robust for different aspiration levels if they are homogeneous [34].

The theorem ensures that the criterion to favor strategy *A* under weak selection limit is a linear inequality, which is independent of all the aspirations. Based on the theorem, the coefficients of the inequality with heterogeneous aspirations are calculated via that with homogeneous aspirations instead. It paves the way to obtain the criterion analytically based on the mean-field approximation [48] (see section *Calculation of coefficients* S1 File). Note that the calculation of the coefficients itself has shown that the criterion to favor strategy *A* does not depend on the aspiration for homogeneous aspiration (see Eq. (12) in S1 File). Yet the calculation for the homogeneous case cannot be extrapolated to the heterogeneous case. The obstacle is overcome by the theorem, which ensures the equivalence of the criterions between homogeneous and heterogeneous aspirations.

The proof of our theorem is similar to the proof in [24, 27, 49] in the sense that they both are based on weak selection limit and symmetric evolutionary rule. Yet a transition probability for the aspiration dynamics is a function of the products of payoff entry and selection intensity and the products of the aspiration and selection intensity, whereas a transition probability for [24, 27, 49] is a function of the products of payoff entry and selection intensity only. This mathematical difference prevents us from directly applying their results [24, 49], and we overcome the obstacle by introducing the two lemmas in the Method section.

We concentrate on a regular network with degree *d* − 1. It is to ensure that every individual has exactly *d* − 1 neighbors such that every individual is able to play the *d*-player game. In fact, the proof of the theorem applies to a set structured population, in which all the individuals are in club-like sets. Therein, each sets consists of *d* individuals, but the number of sets one belongs to varies from individual to individual. In particular, a regular network with degree *d* − 1 is a set structured population, whose set size is *d* and the number of the sets one belongs to is *d* for all the individuals [50]. Furthermore, Fig 2 shows that the criterion in the theorem also holds for random and scale-free networks across a wide range of distributions of individual aspirations. It suggests that the theorem can be true for a wide range of population structures, which requires investigation in the future.

(Fig 1) indicates that there are games such that the heterogeneity in aspiration does not alter the rank of the abundances for non-weak selection, although our theorem is based on the weak selection limit. It seemingly suggests that the criterion to favor a strategy *A* under strong selection is the same as that under weak selection. Yet, it is not true. Let us consider a multiplayer game whose payoff entries are of order 1. In this case, the payoff is of order 1 for both strategies. Let us assume that there are two types of aspirations in the population, one is positive and the other is negative, which are both of higher order than 1. Under strong selection, individuals with high aspiration are never satisfied with their payoffs, and they switch their strategies all the time. By the symmetry of the updating rule (similar to the proof in Section *The average abundance is* 1/2 *for vanishing selection intensity* in S1 File), the two strategies are equally abundant among individuals with high-aspiration. Yet individuals with low aspiration are always satisfied and they act as zealous individuals who never alter strategies [51, 52]. Thus individuals using strategy *A* are more abundant eventually if individuals using strategy *A* with low aspiration are more abundant than those using strategy *B* with low aspiration in the beginning. It clearly indicates that the criterion under which a strategy is more abundant under strong selection differs from that under weak selection. Therefore there are games such that the ranking of strategies is sensitive to the selection intensity in a population with heterogeneous aspirations. In other words, the criterion in the theorem to favor a strategy in abundance is sensitive to the selection intensity, in line with the mutation-selection dynamics [18, 21].

The criterion under which a strategy is more abundant than the other is robust under aspiration dynamics for weak selection limit. In fact, the criterion holds true for various networks, individualised aspirations, and different ways of payoff collection. A potential way to violate the present criterion is to allow individuals to use different updating functions when using strategy *A* and *B*. In this case, updating of the two strategies is not symmetric anymore. And the two strategies can be different in abundance even when all of the payoff entries in the table are the same, violating Lemma 2, which is crucial for the theorem. A study along this way is undergoing.

To sum up, in the limit of weak selection, we show that the heterogeneity in aspiration does not alter the evolutionary outcome compared with the homogeneous case. Our result not only shows a striking difference between comparison based and self-evaluation based rules, but also provides a novel way to compute the aspiration dynamics.

## Supporting information

S1 File Individualised aspiration dynamics. (PDF)

S2 File Table of personal aspiration values in simulations. In the table, personal aspiration values for different simulations are listed. For example, the second column (Column B) shows aspiration values of all the individuals indexed from 1 to 100 and it corresponds to Set 1 of the uniform distribution on the interval [0, 1] used in Figs (1) and (3) (red circles in the left panels).
(XLSX)
