# Supplementary material for: Individualised aspiration dynamics: Calculation by proofs
Source: PLoS Comput Biol. 2018 Sep 25;14(9):e1006035. doi: 10.1371/journal.pcbi.1006035 (PMC6177198; doi:10.1371/journal.pcbi.1006035)
Supplement: S1 File — (PDF) [file pcbi.1006035.s001.pdf]

Supplementary Methods:
Individualised aspiration dynamics: Calculation by proofs

Bin Wu[1*], Lei Zhou[2,3]

1 School of Sciences, Beijing University of Posts and Telecommunications, Beijing 100876, China

2 Center for Systems and Control, College of Engineering, Peking University, Beijing 100871, China

3 Department of Ecology and Evolutionary Biology, Princeton University, Princeton, New Jersey 08544, USA

*** Correspondence author: bin.wu@bupt.edu.cn**

Proofs

The theorem states that it is a linear inequality of payoffs that is the condition under which one strategy is more abundant than the other for aspiration dynamics with heterogeneous expectations. The proof of the theorem is placed in the main text, since it is our main result. Yet the proof of the theorem resorts to the following three statements in this section. Here we outline the proof of the theorem: Firstly, with the aid of Section The average abundance is $1/2$ for vanishing selection intensity, the condition under which one strategy is more abundant than the other under weak selection limit is equivalently transformed to the condition under which one strategy is more abundant than its neutral case. Secondly, Section The criterion is a linear inequality of payoffs and aspirations indicates that the condition is a linear inequality of both payoffs and aspirations. Finally, Section The average abundance is $1/2$ for neutral mutants for any selection intensity indicates that the inequality mentioned in Section The criterion is a linear inequality of payoffs and aspirations has no aspirations at all, which completes the proof of the theorem. Herein Section The average abundance is $1/2$ for neutral mutants for any selection intensity is the cornerstone of the proof of the theorem.

In tphe following, we show the proofs of the three statements which are crucial for the theorem in the main text.

The average abundance is $1/2$ for vanishing selection intensity

Statement: If the selection intensity vanishes, i.e., $\beta = 0$, the average abundance of strategy A is one half for all individualised aspirations and aspiration-based decision making functions.

Proof: Denote q_i as the state of individual i, where i ranges from 1 to N. Let q_i be 1 if it is of strategy A and 0 otherwise. Thus q_i is a random variable. The abundance of individuals using strategy A is $\sum_{i=1}^{N} q_i/N$. And the average abundance of strategy A is given by $\sum_{i=1}^{N} E[q_i]/N$ [1].

Individual i changes its strategy from A to B refers to the case that q_i changes from 1 to 0. Based on the aspiration dynamics, this happens if individual i is selected (with probability $1/N$), and it switches to the other strategy with probability $g_i(\beta(e_i - \pi_i))$. Herein, e_i and π_i are the aspiration and current payoff of individual i, respectively. In particular, when $\beta = 0$, this probability is $g_i(0)$. Note that the probability is independent of the payoff and the aspiration level. The transition matrix for the state of individual i is

$$\begin{array}{c} \\ 1 \\ 0 \end{array} \begin{array}{c} \quad 1 \qquad\qquad\qquad 0 \\ \begin{pmatrix} 1 - \frac{1}{N} g_i(0) & \frac{1}{N} g_i(0) \\ \frac{1}{N} g_i(0) & 1 - \frac{1}{N} g_i(0) \end{pmatrix} \end{array}. \tag{1}$$

Since $g_i(0) > 0$, the Markov chain is irreducible and aperiodic. Thus there is a unique stationary distribution. It is given by the left eigenvector of 1 of the above Matrix (1), i.e., $(\frac{1}{2}, \frac{1}{2})$. Then $E[q_i] = 1/2$ when time evolves sufficiently long for all i. Thus the average abundance of strategy A is $\sum_{i=1}^{N} E[q_i]/N = 1/2$. Furthermore, this is true for all the aspiration levels and all the decision making functions.□

Remark: If the selection intensity is vanishing, neither payoff nor aspiration is at work when strategy updating occurs. It resembles the neutral drift, resulting in the equal abundance between strategy A and B.

The criterion is a linear inequality of payoffs and aspirations

Lemma 1 *For the decision making function g_i with $0 < g_i < 1$ and positive derivative on the real line $g_i' > 0$ $(i = 1, 2, ..., N)$, under weak selection limit, there exist parameters α_k, ω_k $(k = 0, 1, ..., d-1)$ and*

ϕ_i $(i = 1, 2, ..., N)$, *which are neither dependent on payoff entries nor on aspiration levels, such that if*

$$\sum_{k=0}^{d-1} \alpha_k a_k + \sum_{k=0}^{d-1} \omega_k b_k + \sum_{i=1}^{N} \phi_i e_i > 0, \tag{2}$$

then strategy A is more abundant than strategy B.

Proof: Firstly, based on Section The average abundance is $1/2$ for vanishing selection intensity, the average abundance of each strategy is exactly one half when the selection intensity is zero. It is true for any individualised aspirations and any decision making functions. Thus, it is true for the decision making function g_i with $0 < g_i < 1$ and positive derivative $g_i' > 0$. Therefore, the condition under which one strategy is more abundant than the other is equivalent to that one strategy is more abundant than its neutral case.

Secondly, let us denote the i_{th} digit as 1 if individual i adopts strategy A and 0 otherwise. The state of aspiration dynamics on a network as a Markov chain is the binary code with N digits. Thus the state space of the underlying Markov chain is of size 2^N. The transition probability is zero unless the two states differ in at most one digit. Without loss of generality, we assume it is individual i that is trying to update its strategy. The transition probability to another different state is $\frac{1}{N} g_i(\beta(e_i - \pi_i))$. Here e_i is the aspiration and π_i is the current payoff of individual i. Since g_i is differentiable at $\beta = 0$, the transition probability is differentiable at $\beta = 0$. And the first-order derivative of the transition probability with respect to β at $\beta = 0$ is $\frac{1}{N} g_i'(0)(e_i - \pi_i)$. Furthermore, the payoff of individual i, i.e., π_i, is always a linear combination of all the payoff entries, no matter what strategy it is using. This is true for both accumulated and averaged payoffs. Thus the first-order derivative of any transition probability is a linear combination of a_k and b_k $(k = 0, 1, ..., d-1)$ and e_i $(i = 1, 2..., N)$. In other words, the first-order derivative of any transition probability with respect to selection intensity is in the form of $\sum_k (\tilde{\alpha}_k a_k + \tilde{\omega}_k b_k) + \sum_i \tilde{\phi}_i e_i + 0$. It is a linear combination of a_k, b_k and e_i with constant term 0. That is to say, there is no term that does not contain payoff entries or aspiration levels. Note that the coefficients $\tilde{\alpha}_k s, \tilde{\omega}_k s$ and $\tilde{\phi}_i s$ are Taylor coefficients. They are independent of both payoff entries and aspiration levels. In fact, they are only dependent on the decision making functions g_is, the population size and the underlying population structure.

Thirdly, the stationary distribution κ_s $(s \in S)$ is a rational function of transition probabilities. This rational function has no constant terms in both its denominator and numerator. That is to say, there is no term in the numerator (denominator) that does not contain transition probabilities. This result is similar to that in [2, 3]. Here we provide an alternative proof: The stationary distribution κ fulfills the linear equation $\kappa(P - I) = 0$, where P is the transition probability matrix and I is the identity matrix of order 2^N. On the

one hand, the adjugate matrix of $P-I$, $\mathrm{adj}(P-I)$, satisfies $\mathrm{adj}(P-I)(P-I)=\det(P-I)I$ by Cramer's rule. For stochastic matrix P, the row sum is always one. Thus $\det(P-I)=0$. In particular, the first row of matrix $\mathrm{adj}(P-I)$ fulfills $\mathrm{row}_1(\mathrm{adj}(P-I))(P-I)=0$. On the other hand, the stationary distribution is unique for the ergodic Markov chain. Thus κ is $\mathrm{row}_1(\mathrm{adj}(P-I))$ after normalization. κ_s turns out to be a fraction whose numerator is $\mathrm{row}_{1s}(\mathrm{adj}(P-I))$ and whose denominator is $\sum_{s\in S}\mathrm{row}_{1s}(\mathrm{adj}(P-I))$. For $P-I$, each off-diagonal entry of $P-I$ is either in the form of $\frac{1}{N}g_i(\beta(e_i-\pi_i))$ or zero, while each diagonal entry of $P-I$ is in the form of $-\frac{1}{N}\sum_{i=1}^{N}g_i(\beta(e_i-\pi_i))$. In other words, each entry of $P-I$ is a linear combination of (updating function) g_is. Note that the numerator of κ_s, i.e., $\mathrm{row}_{1s}(\mathrm{adj}(P-I))$, is a determinant of a sub square matrix of $P-I$. And it is a sum of products of the entries in $P-I$ with no additional constant terms. By the same argument, the denominator of κ_s is also a sum of products of the entries in $P-I$. In other words, the stationary distribution κ_s is a rational function of all the transition probabilities. Expanding the denominator and numerator of κ_s to the first order with respect to the selection intensify yields

$$\frac{l_0+l_1\beta+o(\beta)}{w_0+w_1\beta+o(\beta)}, \tag{3}$$

and the first-order derivative of the stationary distribution κ_s is $\frac{l_1w_0-w_1l_0}{w_0^2}$. Since w_0 and l_0 are based on the transition probabilities under the neutral selection, they are not dependent on the payoff entries or on the aspiration levels. Meanwhile, w_1 (l_1) is a linear combination of the first-order derivatives of transition probabilities. It is thus a linear combination of e_i ($i=1,2...,N$) and a_k and b_k ($k=0,1,...,d-1$) as discussed in the previous paragraph. This leads to that the first-order derivative of the stationary distribution, $\kappa_s'(0)$, is linear in e_i ($i=1,2...,N$) and a_k and b_k ($k=0,1,...,d-1$), and there is no constant terms.

Denote $|s|$ as the number of strategy A individuals in state s, we now have $\sum_{s\in S}|s|\kappa_s'(0)>0$ if a linear inequality in e_i ($i=1,2...,N$), a_k, and b_k ($k=0,1,...,d-1$) is fulfilled. In other words, there exist parameters α_k, ω_k ($k=0,1,...,d-1$) and ϕ_i ($i=1,2,...,N$), such that if

$$\sum_{k=0}^{d-1}\alpha_k a_k+\sum_{k=0}^{d-1}\omega_k b_k+\sum_{i=1}^{N}\phi_i e_i>0, \tag{4}$$

then strategy A is more abundant than strategy B. Furthermore, the coefficients are neither dependent on the payoff entries nor the aspiration levels. □

Remark: The proof consists of two parts. One is to make an equivalence between the original question

with a new one, i.e., whether the selection intensity increases the abundance of a strategy. The other part makes use of perturbation analysis to arrive at the criterion. In particular, there are few constraints on the decision-making function g_i, but differentiability. This is to ensure that the average abundance is differentiable around $\beta = 0$, such that Taylor expansion works.

The average abundance is $1/2$ for neutral mutants for any selection intensity

Lemma 2 *If all the payoff entries are the same, i.e., there exists a constant h such that $a_k = b_k = h$ for all $k = 0, 1..., d-1$, then the average abundance of strategy A is one half for all possible individualised aspirations, all aspiration-based decision making functions fulfilling $g_i(0) > 0$ ($i = 1, 2, ..., N$), and all selection intensities.*

Proof: Denote q_i as the state of individual i, where i ranges from 1 to N. Let q_i be 1 if it is of strategy A and 0 otherwise. Thus the abundance of individuals using strategy A is $\sum_{i=1}^{N} q_i/N$. And the average abundance of strategy A is given by $\sum_{i=1}^{N} E[q_i]/N$.

q_i changes from 1 to 0, if individual i is selected (with probability $1/N$), and it switches to strategy B with probability $g_i(\beta(e_i - \pi_i))$, where e_i and π_i are the aspiration and current payoff of individual i. In particular, when all the payoff entries are the same, every individual is the same in payoff no matter what strategy it is using and what the configuration of its neighborhood is. It implies that there exists h such that $\pi_i = h$ for all i. Therefore the probability is $g_i(\beta(e_i - h))$ for all the possible aspirations and all decision making functions g_i. In this way, we obtain the following transition matrix for the state of individual i

$$\begin{array}{c} \\ 1 \\ 0 \end{array} \begin{array}{c} \begin{array}{cc} \quad 1 \quad\quad\quad\quad\quad & \quad\quad 0 \end{array} \\ \begin{pmatrix} 1 - \frac{1}{N} g_i(\beta(e_i - h)) & \frac{1}{N} g_i(\beta(e_i - h)) \\ \frac{1}{N} g_i(\beta(e_i - h)) & 1 - \frac{1}{N} g_i(\beta(e_i - h)) \end{pmatrix} \end{array}. \tag{5}$$

Since $g_i(0) > 0$, the Markov chain is irreducible and aperiodic. Thus there is a unique stationary distribution. It is given by the left eigenvector of 1 of Matrix (8), i.e., $(\frac{1}{2}, \frac{1}{2})$. Then $E[q_i] = 1/2$ when time evolves sufficiently long for all i. Thus the average abundance of strategy A is $\sum_{i=1}^{N} E[q_i]/N = 1/2$. Furthermore, this is true for all the aspiration levels and all the decision making functions.□

Remark: The proof is similar to that in Section The average abundance is $1/2$ for vanishing selection intensity. In fact, if all the payoff entries are the same, it is similar to the neutral mutants in population

genetics. Thus, the evolution of strategy for any individual is effectively independent on the others. The critical assumption $g_i(0) > 0$ ensures that every individual has a stationary distribution. By symmetry of the transition probability of the Markov chain, the abundances of the two strategies are equal. Furthermore, it is true for any selection intensity.

Estimating coefficients

Non-negativity of the coefficients

We are going to show i) $\sigma_k \geq 0$ and ii) $\sum_{k=0}^{d-1} \sigma_k > 0$. These indicate that all the coefficients are non-negative, and at least one of those coefficients is positive.

$\sigma_k \geq 0$.

For any $k \in \{0, 1, 2..., d-1\}$, let us choose the payoff table with $a_k = 1$ and all the rest being zero. The theorem in the main text indicates that the first-order derivative of the abundance of strategy A is proportional to σ_k. Thus σ_k is non-negative if and only if strategy A is greater than or equal with strategy B in abundance. In the following, we make use of the equivalence to prove that σ_k is non-negative.

Let us choose an arbitrary individual $i \in \{1, 2, ..., N\}$, and consider the Markov chain with the state space $\{0, 1\}$. Let us denote $x_i(t)$ as the probability with which individual i adopts strategy A at time t. The evolution of $x_i(t)$ is approximated by

$$\frac{d}{dt}x_i(t) = (1 - x_i(t))\frac{1}{N}g_i(\beta(e_i - \pi_i^B(t))) - x_i(t)g_i(\beta(e_i - \pi_i^A(t))) \tag{6}$$

based on the mean-field method. Here $\pi_i^A(t)$ and $\pi_i^B(t)$ are payoffs for individual i, if it is of strategy A and B. It is time dependent due to the strategy adjustment and composition of its neighbourhood. Yet we have that $\pi_i^A(t) \geq 0$ and $\pi_i^B(t) = 0$ taking into account our payoff table. Further, g_i is an increasing function, we have that

$$\begin{aligned}\frac{d}{dt}x_i(t) &\geq (1 - x_i(t))\frac{1}{N}g_i(\beta e_i) - x_i(t)\frac{1}{N}g_i(\beta e_i) \\ &= \frac{1}{N}g_i(\beta e_i)(1 - 2x_i(t))\end{aligned} \tag{7}$$

For the system $\dot{y} = \frac{1}{N}g_i(\beta e_i)(1 - 2y)$, $y^* = 1/2$ is a global stable equilibrium. This indicates that

$\lim_{t\to+\infty} y(t) = 1/2$. Based on comparison principle of differential system [4], we obtain that $x_i(t) \geq y(t)$ for all $t > 0$. Thus $\lim_{t\to+\infty} x_i(t) \geq 1/2$. The average abundance of strategy A is the sum of those probabilities. Therefore strategy A is greater than or equal to one half in abundance. This implies that $\sigma_k \geq 0$ for all $k \in \{0, 1, 2..., d-1\}$.

$\sum_{k=0}^{d-1} \sigma_k > 0$.

In the following we address that the sum of all the coefficients are positive. Let us consider a payoff table with $a_k = 1$ and $b_k = 0$ for all $k \in \{0, 1..., d-1\}$. On the one hand, the first-order derivative of the average abundance of strategy A is $\sum_{k=0}^{d-1} \sigma_k(a_k - b_{d-1-k}) = \sum_{k=0}^{d-1} \sigma_k$ by the theorem in the main text.

On the other hand, for such a payoff table, the payoff of an individual using strategy A is 1, which is independent on its neighbor configuration. Similarly, the payoff of an individual using strategy B is always 0. Denote the state of individual i as 1 and 0, if it is of strategy A and B, respectively. The transition matrix, which is homogenous in time, is given by

$$\begin{array}{c c} & \begin{array}{cc} 1 & 0 \end{array} \\ \begin{array}{c} 1 \\ 0 \end{array} & \begin{pmatrix} 1 - \frac{1}{N} g_i(\beta(e_i - 1)) & \frac{1}{N} g_i(\beta(e_i - 1)) \\ \frac{1}{N} g_i(\beta e_i) & 1 - \frac{1}{N} g_i(\beta e_i) \end{pmatrix} \end{array}. \tag{8}$$

Since $g_i(0)g'(0) > 0$, $g_i(\beta e_i) > 0$ for weak selection. This implies that the above Markov chain is irreducible and aperiodic. Thus there exists a stationary distribution, given by $\frac{1}{g_i(\beta e_i) + g_i(\beta(e_i - 1))}(g_i(\beta e_i), g_i(\beta(e_i - 1)))$. Since g_i is increasing and selection intensity $\beta > 0$, we have that $g_i(\beta(e_i - 1)) < g_i(\beta e_i)$, for all selection intensity. This yields that state 1 is strictly greater than state 0 in probability. The abundance of strategy A is simply the sum of those probabilities, thus strategy A is more abundant that strategy B. This is equivalent with the fact the first-order derivative of the average abundance of strategy A is positive, yielding $\sum_{k=0}^{d-1} \sigma_k > 0$ by the theorem.

Calculation of coefficients

The method we used here is similar to the SI in [5]. The basic idea is to employ pair-approximation to obtain a deterministic equation, and apply the perturbation theory to estimate the coefficients.

An individual, namely i, is selected randomly from the entire population. This individual plays a $d-$player game with all of its neighbours. And it gets payoff π_i. It switches to the other strategy with probability based

on the payoff difference between its payoff and its aspiration, i.e., $[1+\exp(-\beta(e_i-\pi_i))]^{-1}$. Here e_i is the aspiration of the focal individual. In the following we assume $e_i = e$ for all $i = 1, 2..., N$. And we try to calculate the structure coefficients σ_ks ($k = 0, 1..., d-1$) in this case.

Let p_A and p_B denote the frequencies of A and B in the population. Let p_{AA}, p_{AB}, p_{BA} and p_{BB} denote the frequencies of AA, AB, BA and BB pairs. We make use of these six frequencies to approximate that of triplets and higher order moments. Let $q_{X|Y}$ denote the conditional probability to find an X-player given that the adjacent node is occupied by a Y-player ($X, Y \in \{A, B\}$).

Further the following identities hold

$$\begin{aligned} p_A + p_B &= 1, \\ q_{A|X} + q_{B|X} &= 1, \\ p_{XY} &= q_{X|Y} \times p_Y, \\ p_{AB} &= p_{BA}. \end{aligned} \tag{9}$$

It indicates that the system is determined by two independent variables, say p_A and p_{AA}.

Updating a B-player.

Concerning the change of p_A via updating a player using strategy B. Firstly a strategy B individual is selected randomly (with probability p_B). The likelihood with which it has k_A strategy A neighbours and k_B strategy B neighbours is $\binom{d-1}{k_A} q_{A|B}^{k_A} q_{B|B}^{k_B}$. Here $k_A + k_B = d - 1$. Secondly, this strategy B individual switches to strategy A with probability $[1+\exp(-\beta(e-b_{k_A}))]^{-1}$. Therefore, p_A increases by $1/N$ with probability

$$\text{Prob}\left(\triangle p_A = \frac{1}{N}\right) = p_B \sum_{k_A+k_B=d-1} \binom{d-1}{k_A} q_{A|B}^{k_A} q_{B|B}^{k_B} \frac{1}{1+e^{-\beta(e-b_{k_A})}}. \tag{10}$$

Regarding the change of p_{AA} via updating a B-player, if a strategy B individual who has k_A strategy A neighbours is selected and switches its strategy to A, the number of $AA-$pairs increases by k_A. Therefore p_{AA} increases by $k_A/((d-1)N/2)$, where $(d-1)N/2$ is the total number of the links in the population. Therefore

$$\text{Prob}\left(\triangle p_{AA} = \frac{2k_A}{(d-1)N}\right) = p_B \binom{d-1}{k_A} q_{A|B}^{k_A} q_{B|B}^{k_B} \frac{1}{1+e^{-\beta(e-b_{k_A})}}. \tag{11}$$

Updating an A-player.

Concerning the change of p_A via updating an A-player, firstly a strategy A individual is selected randomly (with probability p_A). The likelihood that it has k_A strategy A neighbours and k_B strategy B neighbours is given by $\binom{d-1}{k_A} q_{A|A}^{k_A}\, q_{B|A}^{k_B}$. Secondly, this strategy A individual switches to strategy B with probability $[1+\exp(-\beta(e-a_{k_A}))]^{-1}$. Therefore, p_A decreases by $1/N$ with probability

$$\text{Prob}\left(\triangle p_A = -\frac{1}{N}\right) = p_A \sum_{k_A+k_B=d-1} \binom{d-1}{k_A} q_{A|A}^{k_A}\, q_{B|A}^{k_B}\, \frac{1}{1+e^{-\beta(e-a_{k_A})}}. \tag{12}$$

Regarding the change of p_{AA} via updating an A-player, a strategy A individual is selected (with probability p_A), further it has k_A strategy A neighbours (with probability $\binom{d-1}{k_A} q_{A|A}^{k_A}\, q_{B|B}^{k_B}$). Then it switches to strategy B with probability $[1+e^{-\beta(e-a_{k_A})}]^{-1}$. The number of $AA-$pairs decreases by k_A. Therefore

$$\text{Prob}\left(\triangle p_{AA} = -\frac{2k_A}{(d-1)N}\right) = p_A \binom{d-1}{k_A} q_{A|A}^{k_A}\, q_{B|A}^{k_B}\, \frac{1}{1+e^{-\beta(e-b_{k_A})}}. \tag{13}$$

Pair approximation.

Let us rescale the time step to dt at which a strategy updating happens. Thus

$$\begin{aligned}
\dot{p}_A &= \frac{1}{N}\,\text{Prob}\left(\triangle p_A = \frac{1}{N}\right) - \frac{1}{N}\,\text{Prob}\left(\triangle p_A = -\frac{1}{N}\right) \\
&= \frac{1}{2N}(p_B - p_A) \\
&+ \frac{\beta}{4N}\left[e(p_B-p_A) + \sum_{k_A=0}^{d-1}\binom{d-1}{k_A}\left(p_A q_{A|A}^{k_A} q_{B|A}^{k_B} a_{k_A} - p_B q_{A|B}^{k_A} q_{B|B}^{k_B} b_{k_A}\right)\right] + O(\beta^2)
\end{aligned} \tag{14}$$

and

$$\begin{aligned}
\dot{p}_{AA} &= \sum_{k_A=0}^{d-1}\frac{2k_A}{(d-1)N}\,\text{Prob}\left(\triangle p_{AA} = \frac{2k_A}{(d-1)N}\right) - \sum_{k_A=0}^{d-1}\frac{2k_A}{(d-1)N}\,\text{Prob}\left(\triangle p_{AA} = -\frac{2k_A}{(d-1)N}\right) \\
&= \dot{p}_{AA}|_{\beta=0} + O(\beta) \\
&= \frac{1}{N}(p_{AB} - p_{AA}) + O(\beta)
\end{aligned} \tag{15}$$

Approximating the coefficients.

Based on Eq. (14), p_A is approximately the same as p_B as the system is in the stable state under weak selection limit. The difference is of the first order of selection intensity. Let the frequency of strategy A in the steady state be

$$p_A^* = \tfrac{1}{2} + Q\beta + O(\beta^2) \tag{16}$$

where Q is the Taylor coefficient. Then

$$p_B^* = \tfrac{1}{2} - Q\beta + O(\beta^2) \tag{17}$$

due to the normalisation condition $p_A + p_B = 1$. Based on Eq. (15), p_{AA} is approximately captured by p_{AB} in the stationary state under weak selection limit, $\beta \ll 1$. The difference is of the first-order of the selection intensity. Taking into account Eqs. (9) and the identity $p_{AB} + p_{AA} + p_{BA} + p_{BB} = 1$ yields the frequency for pairs

$$p_{XY}^*(\beta) = \frac{1}{4} + O(\beta), \tag{18}$$

where $X, Y \in \{A, B\}$. Thus we have

$$p_{X|Y}^*(\beta) = \frac{1}{2} + O(\beta), \tag{19}$$

for all $X, Y \in \{A, B\}$.

Inserting Eqs. (16) (17) and(19) into Eq.(14) leads to

$$\dot{p}_A = \frac{\beta}{N}\left(\frac{1}{2^{d+2}}\sum_{k_A=0}^{d-1}\binom{d-1}{k_A}(a_{k_A} - b_{k_A}) - Q\right) + O(\beta^2) \tag{20}$$

In the steady state, we have that $\dot{p}_A = 0$, thus

$$Q = \frac{1}{2^{d+2}}\sum_{k_A=0}^{d-1}\binom{d-1}{k_A}(a_{k_A} - b_{k_A}). \tag{21}$$

Considering $p_A^* = \frac{1}{2} + Q\beta + o(\beta)$p under weak selection, $p_A^* > 1/2$ if Q is positive. Thus if

$$\sum_{k_A=0}^{d-1} \binom{d-1}{k_A} (a_{k_A} - b_{k_A}) > 0, \tag{22}$$

strategy A is more abundant than that of strategy B. Therefore, we have that

$$\sigma_{k_A} = \binom{d-1}{k_A}. \tag{23}$$

Finally, we explicitly obtain the structure coefficients. And these coefficients are also valid for heterogeneous aspiration levels by our theorem.

Estimating the coefficients via simulation

Our theorem indicates that a simple linear inequality of payoff entries suffices to identify which strategy is more abundant. In fact, the linear combination is proportional to the first-order derivative of the average abundance. In other words, the average abundance of strategy A, p_A, is approximately $\frac{1}{2}+Q^*\beta\sum_{k=0}^{d-1}\sigma_k(a_k - b_{d-1-k})$. Here Q^* is a positive constant which is independent of payoff entries.

In particular, for the following payoff matrix (table), the average abundance of strategy A, $p_A(x, y, z)$, on

Number of coplayers with strategy A	0	1	2
A	x	y	z
B	0	0	0

,

the ring with aspiration dynamics is approximately

$$\frac{1}{2} + Q^* \left(\sigma_0 x + \sigma_1 y + \sigma_2 z\right) \beta. \tag{24}$$

Under weak selection limit, $\frac{1}{\beta}(p_A(x, y, z) - \frac{1}{2})$ is approximated by $Q^*(\sigma_0 x + \sigma_1 y + \sigma_2 z)$. Thus $\frac{1}{\beta}(p_A(x, y, z) - \frac{1}{2})$ should lead to a linear combination of x, y and z without any constant term or intercept. The coefficients are what we are aiming for up to a rescaling factor Q^*.

Based on the above argument, we generate a set of points satisfying $x \in [-0.5, 0.5]$ with increment 0.1, $z \in [-0.5, 0.5]$ with increment 0.1 and $y \in [-\frac{x+z}{2} - 0.1, \frac{x+z}{2} + 0.1]$ with increment 0.01. Thus there are $11\times 11\times 21 = 2541$ points. Three sets of individualised aspirations are selected from the uniform distribution on $[0, 1]$, which is found in the SI (file 2). For each (x, y, z) in the set, we estimate the average abundance of

strategy A, i.e. $p_A(x,y,z)$, by simulation: For each data point, it is the mean of 200 independent samples. For each sample, we iterate the process based on aspiration for 10^6 generations. The sample of the average abundance of strategy A is obtained by averaging the last 10^5 generations. Then we employ linear regression to estimate $\frac{1}{\beta}(p_A(x,y,z)-\frac{1}{2})$. In other words, we assume that $\frac{1}{\beta}(p_A(x,y,z)-\frac{1}{2})$ is approximated by $\tilde{\sigma}_0 x+\tilde{\sigma}_1 y+\tilde{\sigma}_2 z+$ intercept. We set the confidence level to be 95% for all the estimated coefficients. The estimated intervals are in the form of [EC-ME, EC+ME], where EC stands for the mean of the estimated coefficients, and 2ME are the length of the interval.

References

1. Karlin S, Taylor HMA. A First Course in Stochastic Processes. 2nd ed. London: Academic; 1975.
2. Tarnita CE, Ohtsuki H, Antal T, Fu F, Nowak MA. Strategy selection in structured populations. Journal of Theoretical Biology. 2009;259:570–581.
3. Wu B, Traulsen A, Gokhale CS. Dynamic properties of evolutionary multi-player games in finite populations. Games. 2013;4(2):182–199.
4. Khalil HK. Nonlinear systems. 3rd ed. Pearson Education; 2013.
5. Du J, Wu B, Wang L. Aspiration dynamics in structured population acts as if in a well-mixed one. Scientific Reports. 2015;5(8014).
